# Health Tract, No. 20

**Published:** 1865

**Authors:** 


HEALTH TRACT, No. 20.

### HEALTH WITHOUT MEDICINE.

### A NEW YEAR'S PRESENT FOR YOUR WIFE.

☞Begin with the January Number.☜

SUBSCRIPTIONS RECEIVED

FOR

### Dr. Hall's Journal of Health.

A MONTHLY PUBLICATION.

*"HEALTH IS A DUTY" INCUMBENT ON ALL.*

*Health is a duty.*—When we announced as a starting-point in the first number of our Journal that a man ought to keep well, and being sick was an implied wrong, no doubt it appeared to many rather a rigid doctrine: to wit, that *it is a sin to be sick.* But men of reflection will not be long in coming to the conclusion, that if it is not so in some cases, it is so in a vast number of instances; and a practical man may benefit himself largely, if he be also conscientious, by inquiring, when incapacitated from discharging the duties of life by illness, "*Is it my fault?*" A servant who cuts off his hand to avoid labor, does, certainly, a deliberate wrong to the person to whom he justly owes his labor. And although we may not deliberately make ourselves sick, yet, if it is done through gross inattention or from ignorance, the degree of criminality in the latter is but a short distance from the former.

To use a not uncommon expression, *a man has no business to be sick.* In other words, his being a sick man *is not always a necessity.* People do not get sick *without a cause*, except in rare cases; and that cause is, very generally, within themselves, resulting from inattention, ignorance, or recklessness, either on the part of themselves, their parents, or their teachers. It is a very poor excuse for a man to say that he can not pay a debt—that declaration becomes insulting to the creditor—when that inability is the result of improvidence or actual extravagance. When any man is disabled by sickness from discharging his duty to himself, his family, or to society, the question should at once be, "*Is it from Heaven or of men?*" Not of the former, for it is said *He* does not willingly afflict the children of men; consequently, sickness is not of *His* sending. It is the result of causes within ourselves. In a literal sense, as well as a moral, it is true, "O Israel! thou hast destroyed thyself!" In plainer terms, disease is not sent upon us; we bring it upon ourselves, and, therefore, *health is a duty incumbent on all*

